# Fatty tissue within the maxillary sinus: a rare finding.

**DOI:** 10.1186/1746-160X-2-28

**Published:** 2006-09-04

**Authors:** Paweł Stręk, Olaf Zagólski, Jacek Składzień

**Affiliations:** 1 Department of Otorhinolaryngology, Collegium Medicum, Jagiellonian University, 2 Śniadeckich St., 31-501 Kraków, Poland

## Abstract

**Background:**

We report a rare case of fatty tissue within the maxillary sinus in a 21-years-old woman, with a history of several previous punctures of the maxillary sinus.

**Case presentation:**

Clinical data of the patient was analysed retrospectively. The patient presented with symptoms of left-sided chronic maxillary sinusitis and had undergone several punctures of the left maxillary sinus 18 months earlier. Subsequent to one of the procedures an acute pain in the left orbit lasting a couple of days was noted. Left endoscopic transnasal antrotomy was performed. The maxillary sinus was filled with polypous, chronically inflamed mucous membrane. Upon its removal, the maxillary roof was identified as drawn downwards and covered with normal mucous membrane. Upon dissection of the membrane, adipose tissue filling the zygomatic recess of the sinus was identified and subsequently removed. The maxillary roof was unchanged. Histopatologic examination confirmed the material to be adipose tissue. No short or long term sequelae occurred.

**Conclusion:**

Adipose tissue can be found in the maxillary sinus most commonly when penetrating from surrounding locations. It is our hypothesis that in the reported patient it penetrated from the orbit to the maxillary sinus following puncture. It seems that a hole in the maxillary sinus roof, about 1 mm in diameter, caused by the needle, may have been a portal of entry for the adipose tissue into the maxillary sinus. The discussed case suggests particular care be taken in performing puncture of the maxillary sinus.

## Background

Orbital content herniation into the maxillary sinus is relatively frequent in orbital floor fractures [1]. Soft tissues (inferior rectus muscle and orbital fat) penetrate through the cracks in the orbital floor which results in limited eye movements and diplopia [1]. Magnetic resonance imaging (MRI) is able to demonstrate orbital floor fractures as sensitively as computed tomography (CT), but CT is superior to MRI in showing small and associated fractures; therefore CT remains the imaging modality of choice in the case of orbital fractures and dehiscences [1,2]. Both MRI and CT are not effective in differentiating adipose tissue from oedematous mucous membrane lining the maxillary sinus. We describe and discuss a case of adipose tissue found in the maxillary sinus that might have penetrated from the orbit into the sinus following a diagnostic puncture. Such a case has not previously been reported.

## Case presentation

**Figure 1:**
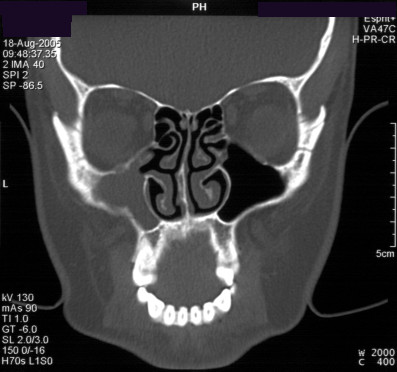
**Preoperative CT scan of the sinuses**. Coronal view demonstrating opacified left maxillary sinus.

**Figure 2:**
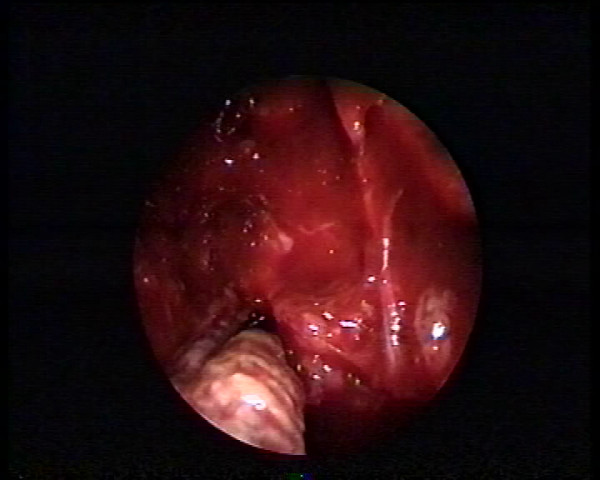
**Endoscopic intraoperative view of the adipose tissue**. The tissue is being removed through the ostium of the maxillary sinus.

**Figure 3:**
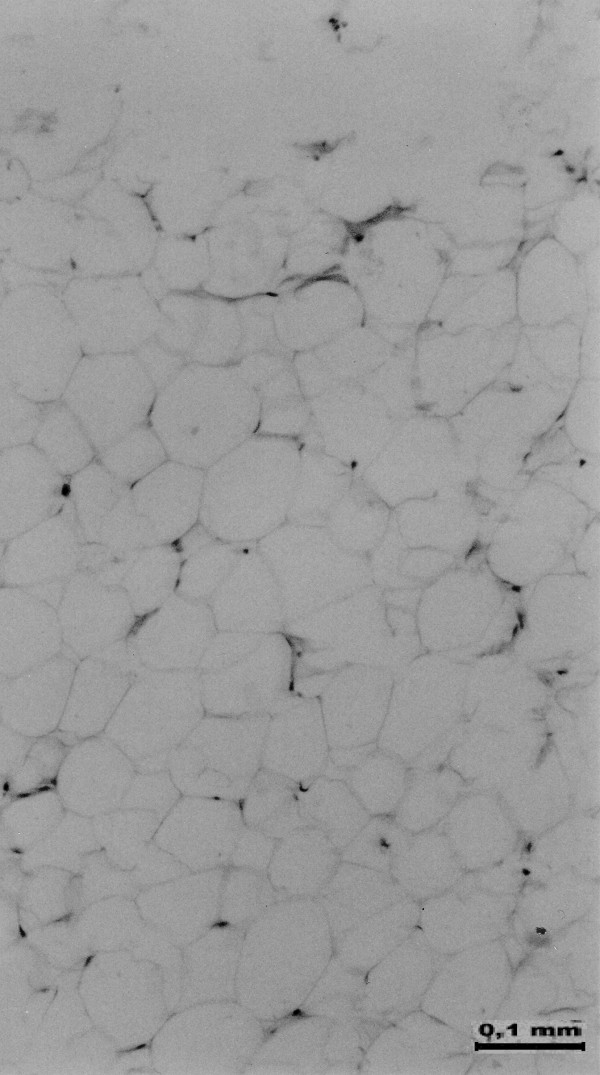
**Histopathological appearance of the fat removed from the maxillary sinus**. H&E staining.

A 21-year old woman presented with a 2-year history of left-sided chronic maxillary sinusitis. The patient had been treated with oral antibiotics and had had several punctures of the left maxillary sinus, performed via inferior nasal meatus, 18 months earlier. One of the procedures was followed by an acute pain in the left orbit lasting a couple of days. CT scans of the sinuses disclosed opacified left maxillary sinus (Figure 1). Left endoscopic antrotomy was performed. The sinus proved to be filled with polypous, chronically inflamed mucous membrane. On removing it, the maxillary roof (orbital floor) was identified as drawn downwards, covered with normal mucous membrane. It was dissected and adipose tissue filling the zygomatic recess of the sinus, 25 mm in diameter, total tissue volume of about 1 cm^3^, was exposed (Figure 2). The tissue was removed. The maxillary roof was normal upon thorough examination and repeated analysis of CT scans. Orbital floor damage was excluded by pressing the left eyeball and simultaneously observing the superior wall of the maxillary sinus in search of inferior rectus muscle movement. The patient had 1 mm enophthalmos in the left eye after surgery. She did not report diplopia. Her left eye movements were not limited in the vertical and horizontal planes. Histopatologic examination of the operative specimen confirmed adipose tissue (fat) (Figure 3). The patient has since been asymptomatic.

## Discussion

Adipose tissue can be found in the maxillary sinus either when penetrating from surrounding locations or in rare cases of fat and adipose tissue tumours [3,4]. In a study of 256 non-epithelial neoplasms involving the nasal cavity, paranasal sinuses and nasopharynx, reported by Fu et al. [5] only two lesions were classified as adipose tissue tumours (one lipoma and one liposarcoma) [5]. However, a lipoma of the maxillary sinus could be a relevant differential diagnosis for the presented case. Normal fat fills the bucca in front of the lateral wall of the maxillary sinus [6] but in young individuals the bone is thick enough and not yet pneumatized so it prevents soft tissue from penetrating into the sinus cavity through dehiscences. In the presented patient none of the punctures were performed through the lateral sinus wall. Adipose tissue is a usual finding in the pterygopalatine fossa [7]. In orbital floor fractures, orbital adipose tissue penetrates into the maxillary sinus [1] through relatively large cracks. The amount of fatty tissue found in the maxillary sinus of our patient correlated with the degree of enophthalmos. It is our hypothesis that the fatty tissue could have penetrated from the orbit into the maxillary sinus through the aperture caused by the puncture needle. Puncture of the maxillary sinus is considered the gold standard for diagnosing bacterial maxillary sinusitis [8]. It is usually performed via the inferior nasal meatus and the needle should be directed at such an angle so as not to interfere with the roof of the maxillary sinus [8]. In the reported patient, the needle could have perforated the superior sinus wall. Data from the available literature and our own experience prove that in lamina papyracea dehiscences or iatrogenic defects subsequent herniation of orbital content into the ethmoids also occurs [2], suggesting that orbital adipose tissue has a tendency to penetrate into surrounding locations. It seems that even a hole of about 1 mm in diameter caused by the needle might have been a portal of entry for the adipose tissue into the maxillary sinus, before the aperture healed. CT scans performed in our patient prior to surgery did not disclose any dehiscence but even low-thickness slices of the CT scan may not detect such a small aperture. It might, however, have healed during the period between the puncture and surgery.

## Conclusion

Adipose tissue can be found in the maxillary sinus most commonly when penetrating from surrounding locations. It is our hypothesis that in the reported patient it penetrated from the orbit to the maxillary sinus. The discussed case suggests particular care should be taken in performing puncture of the maxillary sinus.

## Competing interests

The author(s) declare that they have no competing interests.

## Authors' contributions

PS performed the described operation and participated in the paper design.

OZ conceived the paper design, drafted the manuscript and wrote the text.

JS participated in the paper design.

All authors read and approved the final manuscript.
